# EdgeCrafting: mining embedded, latent, nonlinear patterns to construct gene relationship networks

**DOI:** 10.1093/g3journal/jkac042

**Published:** 2022-02-17

**Authors:** Benafsh Husain, Matthew Reed Bender, Frank Alex Feltus

**Affiliations:** 1 Biomedical Data Science and Informatics Program, Clemson, SC 29631, USA; 2 Department of Genetics and Biochemistry, Clemson University, Clemson, SC 29631, USA; 3 Center for Human Genetics, Clemson University, Greenwood, SC 29646, USA

**Keywords:** biological networks, blob detection, gene expression

## Abstract

The mechanisms that coordinate cellular gene expression are highly complex and intricately interconnected. Thus, it is necessary to move beyond a fully reductionist approach to understanding genetic information flow and begin focusing on the networked connections between genes that organize cellular function. Continued advancements in computational hardware, coupled with the development of gene correlation network algorithms, provide the capacity to study networked interactions between genes rather than their isolated functions. For example, gene coexpression networks are used to construct gene relationship networks using linear metrics such as Spearman or Pearson correlation. Recently, there have been tools designed to deepen these analyses by differentiating between intrinsic vs extrinsic noise within gene expression values, identifying different modules based on tissue phenotype, and capturing potential nonlinear relationships. In this report, we introduce an algorithm with a novel application of image-based segmentation modalities utilizing blob detection techniques applied for detecting bigenic edges in a gene expression matrix. We applied this algorithm called EdgeCrafting to a bulk RNA-sequencing gene expression matrix comprised of a healthy kidney and cancerous kidney data. We then compared EdgeCrafting against 4 other RNA expression analysis techniques: Weighted Gene Correlation Network Analysis, Knowledge Independent Network Construction, NetExtractor, and Differential gene expression analysis.

## Introduction

Biological network construction is a fundamental technique to explore genetic relationships. It has been estimated that any given gene will have direct interactions with 4–8 other genes while being involved in approximately 10 biological functions (Arnone and Davidson, 1997) . Typical biological networks include protein–protein interaction networks (Jeong et al. 2001), signaling networks (Olsen et al. 2006), metabolic networks (Jeong et al. 2000), gene coexpression networks (GCNs) (Ficklin et al. 2017), and gene regulatory networks (GRNs) (Düvel et al. 2010). GCNs have been conventionally constructed using linear or monotonic metrics such as Pearson or Spearman correlation between the expression values for every pair of genes. The network typically comprises of gene–gene interactions, known as edges, that meet a threshold defining significant correlation. Clusters of highly interconnected genes can be identified as modules, thus suggesting relationships between condition-specific, highly correlated genes.

Conventional algorithms such as weighted GCN analysis (WGCNA) (Langfelder and Horvath 2008) are limited by the fact that they can only identify linearly correlated relationships while struggling to differentiate between intrinsic signal and extrinsic noise. To this end, algorithms such as Knowledge Independent Network Construction (KINC) (Ficklin et al. 2017) and NetExtractor (Husain et al. 2020) were developed to capture linear and potentially nonlinear relationships that have traditionally been ignored. Integration of these nonlinear relationships, however, greatly increases the computational complexity of constructing relationship networks. This issue can be compounded by the presence of highly related subpopulations within the sample distribution. In addition, network construction algorithms become particularly unwieldy when a large number of samples are considered on account of the pair-wise correlation analysis.

We propose a novel approach for identifying bigenic, differentially expressed relationships by reconstructing the input gene expression matrices (GEMs) into a binned image-based format. Kernels of this idea were first introduced in EdgeScaping (Husain and Feltus 2019) where this novel representation of gene expression profiles allowed us to utilize a plethora of image analysis modalities previously unexplored in the realm of transcriptomics. EdgeCrafting implements a popular image segmentation technique to identify sub-populations within each correlation network, known as “blob detection.” EdgeCrafting proposes to transform the manner in which transcriptomic data are conventionally represented and the bigenic coexpression relationships are defined. In this report, we apply EdgeCrafting to a human transcriptome compendium and then compare the constructed network to existing linear and nonlinear GCN construction algorithms.

## Materials and methods

### Input data

To implement the various differential gene expression (DGE) analysis algorithms, we utilized the RNA-sequencing (RNAseq) expression values for both “normal” tissue samples and “tumor” tissue samples that can be directly comparable. Two of the larger resources available for gathering RNAseq expression values include panTCGA and panGTEx GEMs (Lonsdale et al. 2013). The panGTEx GEM comprised of 56,202 genes across 11,688 samples, which is split among 53 tissue types. The panTCGA GEM (Hoadley et al. 2018) contained 60,101 genes measured across 11,093 tissue samples, which is split between 33 cancer phenotypes. The primary limitation, however, is that these GEMs cannot be directly compared against each other since they have not be normalized together to ensure the uniformity of sample expression values. Thus, we utilized unified TCGA and GTEx tissue data documented by Wang et al. (2018) for “kidney” datasets. This GEM comprised of 158 normal human kidney samples, 60 chromophobe renal cell carcinoma (KICH) samples, 475 clear cell renal carcinoma (KIRC) samples, and 236 papillary renal cell carcinoma (KIRP). This renormalized, unified GEM contained 19,216 genes measured across a total of 929 samples and was the subject of our EdgeCrafting and comparative analyses.

### WGCNA network construction

One of the algorithms used to compare with EdgeCrafting includes WGCNA (Langfelder and Horvath 2008), an R package for weighted correlation network analysis to identify modules of highly correlated genes. The WGCNA tool package consists of a variety of specific functions for network generation across a broad scope of data types and user requirements. WGCNA is typically used to identify clusters within the constructed network and to further study the relationships between these coexpressed groups of genes. WGCNA uses a preprocessed input matrix of RNAseq data and along with a similarity metric to create an adjacency matrix. A thresholding parameter utilizing scale-free topology is then used to obtain clusters of similarly expressed genes. Despite its wide adoption within the bioinformatics community, this tool suffers a limitation in its inability to capture nonlinear relationships. Another limiting factor is the inability to scale to large GEMs such as panTCGA and panGTEx, so we narrowed our scope to only kidney data to escape this scaling limitation. For the purpose of kidney GEM evaluation, we utilized the WGCNA python wrapper (Greenfest-Allen et al. 2017) with default settings to obtain 32 modules that contained 1,881 nodes (genes). Note that WGCNA does not output edge weight associated to the modules due to its functionality as a clustering algorithm. WGCNA’s purpose is to form clusters of correlated genes rather than rank hierarchies of weight to each node interaction.

### KINC network construction

The KINC (Ficklin et al. 2017) tool aims to mitigate the shortcomings associated with variations in noisy (intrinsic or extrinsic) GEM data. KINC (software package available at http://www.github.com/SystemsGenetics/KINC) analyzes GEM data in a bigenic manner and hypothesizes the existence of multiple modes when searching for condition-specific coexpression relationships. These modes may pertain to the differentially expressed conditions in the gene pair as well as the isolation of expression from intrinsic or extrinsic and statistical noise. KINC utilizes Gaussian mixture models (GMMs) to identify these modes within each bigenic coexpression distribution. For each detected mode, KINC calculates the Spearman correlation and considers only modes containing 30 samples or greater. The correlation threshold was identified using random matrix theory (RMT). Modes with fewer than 30 samples or an Fragments Per Kilobase of transcript per Million mapped reads (FPKM) expression of less than 0.1 were ignored. KINC outputs a correlation matrix along with generating a network file containing edges and metadata describing the network.

### NetExtractor network construction

**Fig. 1.**
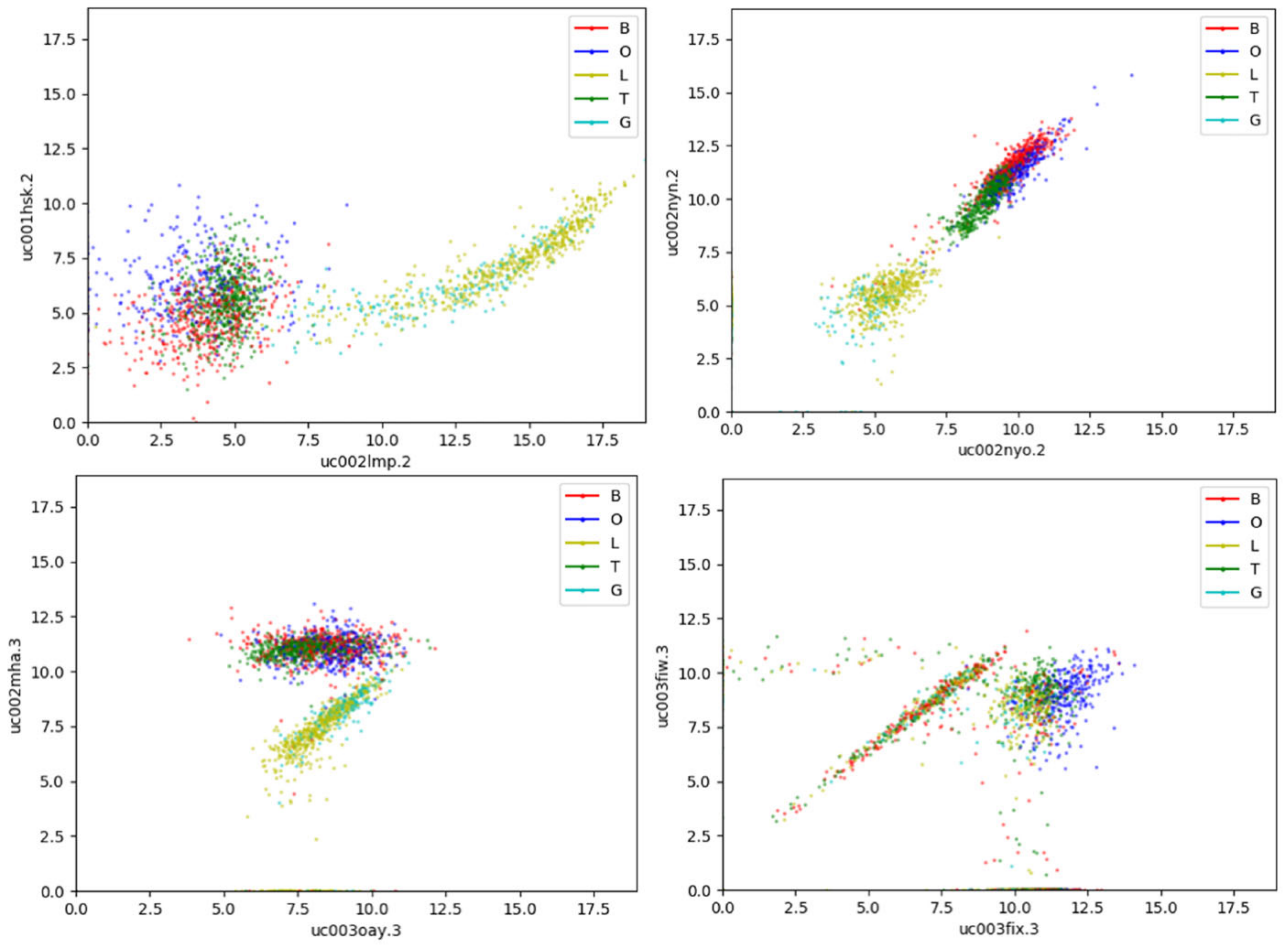
Examples depicting *potentially nonlinear* modes of differential expression patterns for bigenic coexpression data. B, bladder cancer; G, glioblastoma; L, lower-grade glioma; O, ovarian cancer; T, thyroid cancer.

Conventionally, linear metrics such as Spearman or Pearson’s correlation are primarily utilized when defining relationship for bigenic expression data such as depicted by WGCNA, and even though KINC implements GMM to identify several modes of relationships within each gene pair, linearity of coexpression relationship is still an underlying assumption. Examples depicted in Figure 1 for the coexpression distribution of tumor samples, more specifically bladder cancer (BLCA), ovarian cancer (OV), lower-grade glioma (LGG), thyroid cancer (THCA), and glioblastoma (GBM), exhibit the typical relationship NetExtractor aims to capture where cases consisting of multiple modes within bigenic expression data may not always restrict to linearly defined coexpressions. In Fig. 1, it can be observed that samples under different conditions (e.g. GBM and LGG vs BLCA, OV, and THCA) form distinct subpopulations within coexpression depictions and may require nonlinear metrics to be identified. Hence, NetExtractor expands upon the theory of GMM-based KINC network construction by utilizing a nonlinear method of mutual information (MI) to define gene–gene interactions (available at https://github.com/bhusain/NetExtractor.git). MI is a measure of similarity between 2 random variables (RV) where if $\left|U_{i}\right|$ is the number of the samples in module *U_i_*, and $\left|V_{j}\right|$ is the number of the samples in module *V_j_*. The MI between modules *U* and *V* is expressed by:

$$\text{MI}(U,V)=\sum_{i=1}^{\left|U\right|}\sum_{j=1}^{\left|V\right|}\frac{\left|U_{i}\cap V_{j}\right|}{N}\;\text{log}\;\frac{N|U_{i}\cap V_{j}|}{\left|U_{i}||V_{i}\right|}$$

NetExtractor utilizes a Python Scikit-learn package *sklearn.mixture.BayesianGaussianMixture* to estimate the different GMM modes, followed by the estimation maximization algorithm. Python’s Scikit-learn package was also utilized to calculate MI in the range of 0 (no correlation) and 1 (perfect correlation) per subpopulation within a gene-pair edge. The final MI value assigned to each bigenic relationship is the average of all subpopulations. Another threshold applied for gene pairs is mean silhouette coefficient (*S*), which is calculated using the mean of intramodule distance (*a*) as well as the mean nearest module distance (*b*) using the equation (*ba*)/max(*a*, *b*) by implementing the package *sklearn.metrics.silhouettescore*. The score, *S*, quantifies the “tightness” or “similarity” of the detected subpopulation. Since MI is a nonlinear metric where the correlation of 1 RV is dependent on the predictability of the other RV, it is expected to have a large number of gene pairs meet even a reasonably large cutoff threshold; hence, NetExtractor utilized both MI and *S* measures to restrict the number of gene pairs (edges) of interest.

### DGE analysis

A more straightforward and conventionally adopted gene expression analysis technique is the DGE analysis that provides a probability statistic that any given gene will be significantly differentially expressed between 2 phenotypes or conditions. There are several implemented techniques for DGE calculation, but in this paper, we used the Bioconductor package DESeq2 (Love et al. 2014). DESeq2 compares DGE between conditions using the negative binomial probability distribution. To analyze the results of DGE, statistical outliers that represented the most highly differentially expressed genes [both up (positive) and down (negative) regulated] were isolated (*P*_adj_ < 0.0001).

### EdgeCrafting network construction

**Fig. 2.**
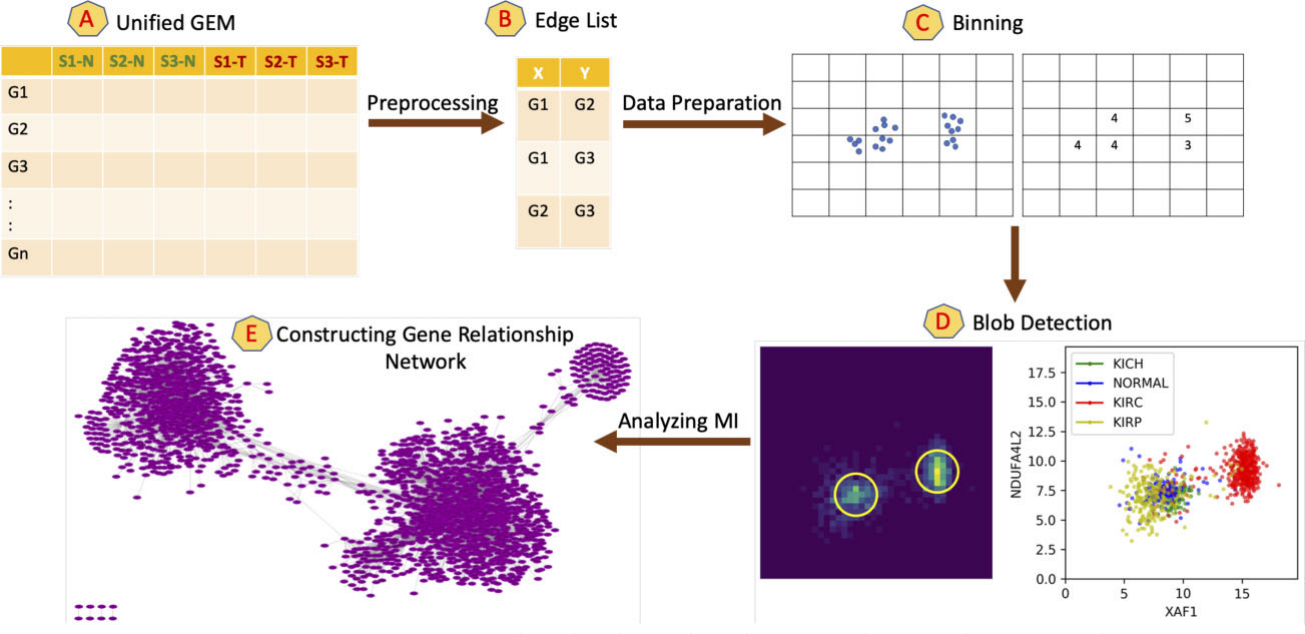
EdgeCrafting workflow: Key stages of algorithm depicted by blocks A–E. Blocks A and B represent the preprocessing of the input GEM. Block C depicts the restructuring of GEM data into an image based format. Block D depicts the implementation of “blob detection” techniques used to identify subpopulations. Block E represents the final constructed gene coexpression network.

The primary contribution of this paper is the implementation of the EdgeCrafting workflow as depicted by Fig. 2. EdgeCrafting attempts to recreate the methodology of KINC and NetExtractor but transforms the data in a novel manner to achieve similarly correlated gene expression networks while reformulating the manner in which we understand and manipulate the input data. In block A (unified GEM) of Fig. 2, we depict the input unified kidney GEM. The output from this block is then used for further preprocessing of data format in block B (edge list) where an edge list is generated as a 2-dimensional RNAseq array for every gene pair. Block C (binning) represents the primary data transformation technique that was first introduced in EdgeScaping (Husain and Feltus 2019).

**Fig. 3.**
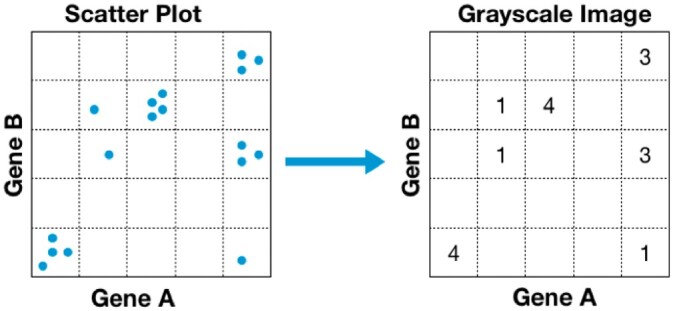
Converting bigenic expression data into an image analytics format. The number of samples that fall into each gene expression bin is counted transforming gene expression RNA-seq data into an image based format.

Figure 3 depicts an example of the binning process, which converts the 2-dimensional array from block B into a grayscale image, where the range of input GEM data determines its dimensions. For example, in our particular case min = 0 (only positive expression levels are considered) and max = maximum expression value within the GEM (e.g. for the kidney GEM the max value was 18.89 FPKM). Hence, the size of the grayscale image is set at 19 × 19, which is then binned into 19 equal sizes per dimension. The value of each bin was calculated by the number of samples assigned to that bin as determined by their expression range. This transforms the conventional gene-pair representation from 2-dimensional sized array of expression data (929 × 2 for unified kidney GEM) of block B to a grayscale image of dimension 19 × 19. EdgeCrafting can also be implemented at different resolution of bin sizes based on the number of samples available as well as the distribution patterns of coexpressions. We tested 3 resolutions at: 1 (19 × 19 bins), 0.5 (38 × 38 bins) and 0.25 (76 × 76 bins), and selected the final bin resolution as 0.5 (38 × 38 bins) that maintained the most fidelity of the original input data.

Having transformed the original data into a format akin to an image, we proceeded to utilize techniques and algorithms that have been developed specifically for image manipulation. In EdgeScaping, clustering algorithms were utilized to group gene-pair edges based on similar expression patterns, whereas the next block of EdgeCrafting (block D) used “blob detection,” a classic segmentation methodology widely adopted in image analysis. A Python skimage package (van der Walt et al. 2014) was utilized to calculate the subpopulation of bigenic sample distributions depicted as “blobs” using 3 blob detection algorithms, namely Laplacian of Gaussian (LoG), Difference of Gaussian (DoG), and Determinant of Hessian (DoH) (Kong et al. 2013). Blobs in EdgeCrafting are similar in concept to the “modes” detected using GMMs in KINC and NetExtractor. Also similar to GMMs, blob detection can be highly sensitive, and there might be cases where “blobs” within an edge are detected but are not necessarily significantly coexpressed.

We calculated the MI for all the samples within the bigenic data to determine an appropriate threshold for network construction. Note that similar to NetExtractor, we also calculated MI per blob and estimated the effective MI per gene edge by averaging MI over all detected modes. Since the network was not significantly altered, we implemented the more straightforward approach of calculating the MI using all the samples within the bigenic coexpression data. Another criteria used for filtering bigenic coexpression date to qualify for inclusion into the final network is that at least 2 or more blob detection techniques (LoG, DoG, or DoH) should detect more than 1 subpopulation, i.e. we restrict to nodes that specifically demonstrate differential expression and are not false positives. Finally, in the last block E (constructing gene relationship network), a network is constructed where nodes represent genes while the edges represent the bigenic coexpressions that meet the thresholding criteria.

## Results

To implement and compare the various GCN construction algorithms we leveraged the unified GTEx and TCGA GEMs published in (Wang et al. 2018). In this report, we limited our analysis to the 19,216 × 929 “kidney” GEM that comprised of 158 normal human kidney samples, 60 KICH samples, 475 KIRC samples, and 236 KIRP samples for all algorithms. The output of all algorithms was then compared based on the detected gene and edge relationship as well as the functionally enriched terms associated with them. Table 1 depicts the nodes and edges detected for each algorithm.

**Table 1.** Number of genes and edges detected with the 5 algorithms.

Algorithm	WGCNA	KINC	NetExtractor	EdgeCrafting	DGE_neg	DGE_pos
Genes	1,881	959	3,772	2,275	6,303	4,557
Edges	Not Applicable	8,913	16,824	7,328	Not Applicable	Not Applicable

### Algorithm comparisons

#### WGCNA

The first algorithm we used to analyze the unified kidney GEM was WGCNA, a software package implemented in R for linear GCN construction. WGCNA comprises of a comprehensive collection of R functions that can be utilized for performing various aspects of weighted correlation network analysis. Cluster detection and analysis is a common functionality provided by WGCNA, where clusters in this context represent *group of genes* that exhibit similar expression patterns. WGCNA typically uses the Pearson or Spearman correlation metric with a user defined threshold to construct the GCN. Along with the restriction of linearity or monotonic relationships, another severe drawback to WGCNA is the lack of scalability to larger GEMs. As the number of samples increases, evaluation of each gene pair becomes increasingly computationally intensive. GEMs that are significantly larger than the unified kidney GEM such as panTCGA and panGTEx reached a bottleneck on the available Palmetto cluster resources and did not reach convergence. Updates to the conventional WGCNA algorithms included using MI as a possible metric along with soft thresholding, but for the purpose of comparing to EdgeCrafting, we limited our analysis to the basic version of the algorithm and only utilize the modules of gene clusters detected. For the purpose of kidney GEM evaluation, we utilized the WGCNA python wrapper (Greenfest-Allen et al. 2017) with default settings to obtain *32 modules* that contained *1,881 nodes (genes)* depicted in Table 1.

#### KINC

**Fig. 4.**
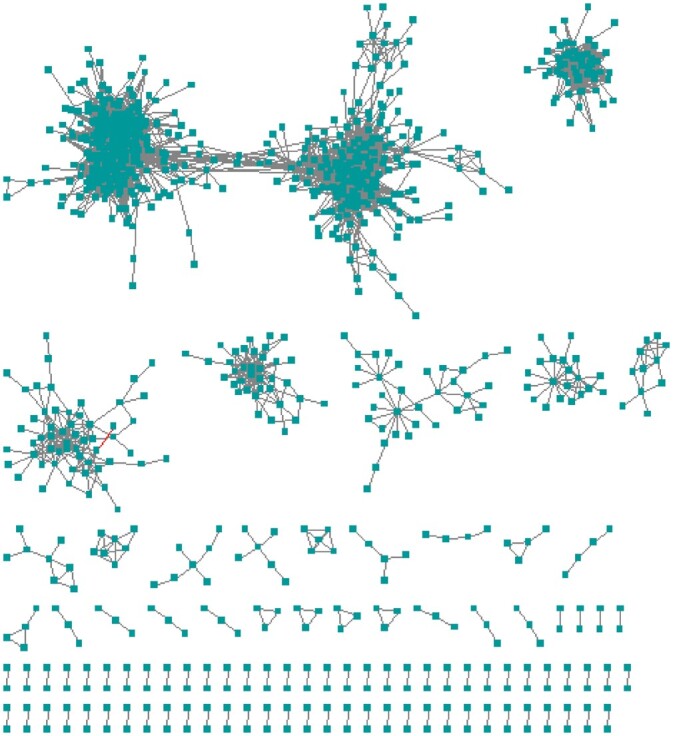
KINC kidney gene relationship network. This network was constructed using KINC and consists of 8,913 edges connected by 959 genes.

Another method we used to compare gene network construction was KINC, which utilized the approach of GMMs to construct a condition-annotated GCN. That approach was specifically designed to address natural extrinsic variation during network construction from mixed input conditions. With the hypothesis that gene expression relationships exhibit modality, the GMMs allow for the identification of multiple modes for each pair-wise gene expression. The correlation significance threshold calculated was 0.859 using RMT, which resulted in a scale-free network with *8,913 edges* connected by *959 genes*. Figure 4 depicts the constructed KINC network with Table 1 compares the KINC output to the other algorithms.

#### NetExtractor

**Fig. 5.**
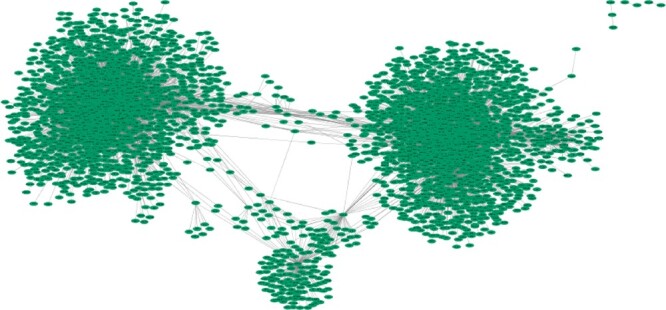
NetExtractor kidney gene relationship network. This network was constructed using NetExtractor and consists of 16,824 edges connected by 3,772 genes.

NetExtractor was designed to transcend the limitations posed by the exclusion of nonlinear bigenic relationships for GCN construction, as well as the problem posed by noisy correlated edges. NetExtractor is a workflow that minimizes the problems of extrinsic noise with the application of GMMs while exploring nonlinear latent relationships using the MI metric. MI predicts the dependence of 1 RV over another RV which is not restricted to linearity between gene expression relationships. NetExtractor was also designed to identify condition-specific correlations and differential expressions in RNAseq data between gene pairs. For the unified kidney GEM, the MI threshold selected was 0.975 while the intercluster score, *S*, was calculated as 0.77. Similar to previously discovered networks, NetExtractor resulted in *16,824 edges* connected by *3,772 genes*. The constructed NetExtractor GCN is depicted by Fig. 5 with Table 1 comparing the NetExtractor output to the other algorithms.

#### DGE analysis

We also compared the GCN construction methods to conventional DGE analysis techniques that utilizes single gene expression values for different condition types as opposed to analyzing bigenic coexpression relationships. DGE analysis in this report was performed using the Bioconductor package DESeq2 (Love et al. 2014). Rather than using normalized estimated expression levels, raw RSEM counts of sequencing reads were used. These datasets were published by Wang et al. alongside the normalized expression values. In this report, we distinguished the set of genes, which have statistically significant positive regulation and the gene set that have a significant negative regulation; hence, these 2 list of genes are mutually exclusive. As documented in Table 1, we obtained *6,303 negatively regulated genes* and *4,557 positively regulated genes*.

#### EdgeCrafting

One of the novel contributions of this report is the implementation of the EdgeCrafting algorithm, which is inspired by the detection of subpopulation modes within bigenic expressions depicted in KINC and NetExtractor. It attempts to address the limitations of data explosion that leads to extensive computational resources with the introduction of calculating subpopulations within sample distributions using nonlinear metrics such as MI. In a process similar to the one described in EdgeScaping, the first step in EdgeCrafting was to rearrange the format of the input GEM into an image-based binned data. To overcome the increasing dimensionality problem that is pervasive in GCN construction as datasets grow in size, we implemented a novel solution of data compression (binning) while still maintaining the feature information per edge (gene pair).

**Fig. 6.**
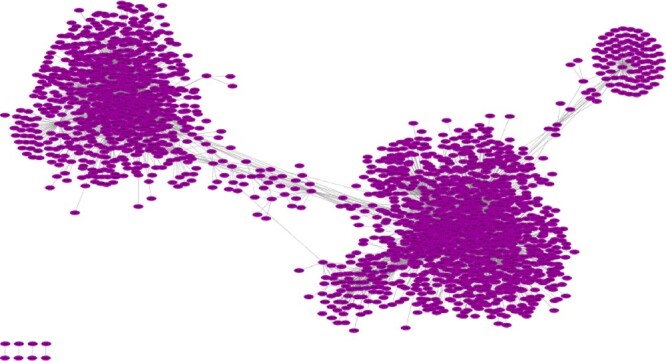
EdgeCrafting kidney gene relationship network. This network was constructed using EdgeCrafting and consists of 7,328 edges connected by 2,275 genes.

We narrowed potential edges to those that depicted 2 or more blobs within at least 2 of the “blob detection” approaches to ensure the blobs represent differentially expressed values between tissue samples and not false positives due to algorithm sensitivity. In a manner similar to NetExtractor, we followed the process of blob detection by calculating the MI of each blob as well as all the samples simultaneously while taking the average to calculate the effective MI score for that particular edge. We further compared this effective MI score to the MI score using all samples in the selected edges and did not find a significant difference hence choosing the latter. The final threshold selected for an edge to be included in the network was 0.97. The resulting extracted network resulted in *7,328 edges* connected to *2,275 genes*. The constructed EdgeCrafting GRN is depicted by Fig. 6 with comparisons to other algorithms listed in Table 1.

An important consideration we addressed in EdgeCrafting is the issue of axis orientation for genes. In the implementation of EdgeScaping, it can be noted that there was no restriction of axis onto which a particular gene falls. This directly impacts the pattern of the image being created. Therefore, during the clustering procedure of EdgeScaping a mirroring effect was present, where a similar pattern is classified as 2 distinct clusters simply because the *x-* and *y*-axes of the particular gene was flipped. This phenomenon was mitigated for EdgeCrafting as we detached orientation from edge selection since blob detection within each image is independent of the orientation of the image. To confirm that we address the shortcoming of orientation, we ran the EdgeCrafting algorithm twice on the same unified kidney GEM, where the second time the *x* and *y*-axes for the genes were flipped. We compared the networks generated in both runs to confirm that orientation does not play a role in edge selection.

**Fig. 7.**
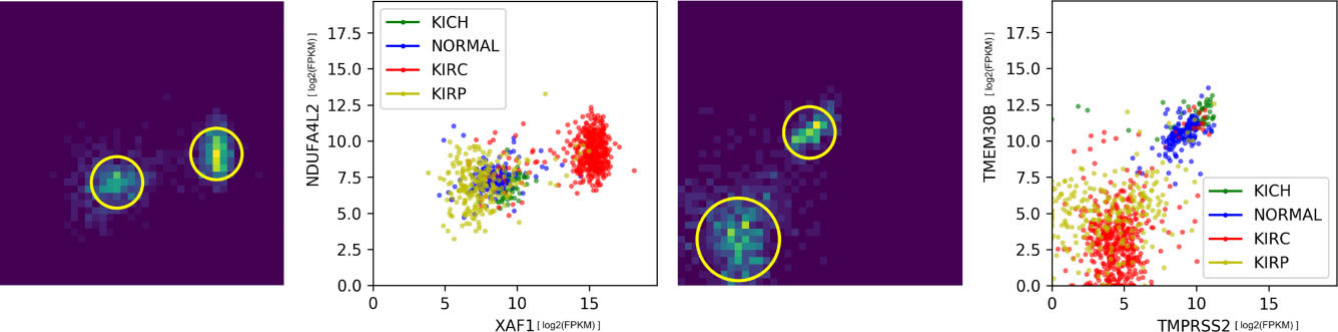
Example EdgeCrafting edges. Left 2 panels: scatterplot of input data between *XAF1* and *NDUFA4L2* genes. The plot depicts the image based representation of the input data along with the detected blobs. Figure also depicts KIRC with a higher differential expression values when compared against the other tissue samples. Right 2 panels: scatterplot of input data between *TMPRSS2* and *TMEM30B* genes. The plot depicts the image based representation of the input data along with the detected blobs. The figure also depicts KIRC and KIRP with a lower differential expression values when compared against the other tissue samples.

An example of EdgeCrafting output is depicted by Fig. 7, where the subfigure on the right is the scatterplot of gene pairs with every sample point colored based on the specific tissue type, i.e. normal, KICH, KIRP, and KIRC tissue samples. The corresponding subfigure on the left represents the detected blobs within an image-based compressed format. The results of EdgeCrafting were observed to maintain the fidelity of data even when compressed into a binned image based format.

#### Overlapping gene and enriched terms

Table 2 lists the number of genes detected per algorithm and their overlap against all the other techniques. It can be observed that the highest overlap of genes among the different algorithm outputs was between the NetExtractor and EdgeCrafting results, reflecting 57% overlap with 2,208 genes in common. This suggests that blob detection results can closely resemble GMM-based techniques for differentially expressed bigenic data. As specific examples that were detected by both EdgeCrafting and NetExtractor, we can observe that for Fig. 7 (left) for genes *NDUFA4L2* vs *XAF1*, 2 blobs were detected with KIRC samples having higher expression values for that gene pair when compared against other tumor or normal tissue samples. Similarly, in Fig. 7 (right), we observe that KIRP and KIRC samples are expressed lower when compared to normal and KICH tissue samples for genes *TMEM30B* vs *TMPRSS2*. Table 2 also indicates that there is a substantial correspondence between WGCNA and KINC algorithm outputs with 35.9% (745) overlapping genes. This also follows our expectations due to the fact that both algorithms search for linearly correlated edges either with or without the presence of subpopulations. Another interesting observation is that NetExtractor and EdgeCrafting share relatively higher percentage of genes with WGCNA than they do with KINC. In a similar trend, it can also be observed that the output of DGE demonstrates a fairly low overlap with WGCNA and KINC when compared against NetExtractor and EdgeCrafting.

**Table 2.** Number of overlapping genes among the 5 algorithms.

	WGCNA	KINC	NetExtractor	EdgeCrafting	DGE_neg	DGE_pos
WGCNA	1,881					
KINC	35.6% (745)	959				
NetExtractor	26% (1,168)	14% (583)	3,772			
EdgeCrafting	25% (832)	16% (457)	57% (2,208)	2,275		
DGE_neg	6.6% (397)	2.6% (143)	18.8% (1,319)	11.1% (686)	6,303	
DGE_pos	12% (879)	8.4% (568)	17.5% (1,502)	14.5% (1,089)	0	4,557

To further compare the networks from all the algorithms, we performed functional enrichment analysis (Chen et al. 2009) on the genes sets from each algorithm and tabulated the results in Tables 3–7. Each of these tables account for the number of enriched terms for all the algorithm with a significance threshold of False Discovery Rate (FDR) *q*-value <0.00001. A stringent significance threshold was set due to the fact that enrichment terms are often significant with a low number of queried genes. Because these algorithms are providing lists with several hundred genes of interest, it is necessary to set a more stringent threshold. It is not uncommon to see FDR *q*-values in the range of 0.001–0.05 for random gene sets of this size. A stringent threshold was set to ensure that the enrichment results were accurate reflections of the biological patterns underlying these insights. Each table also contains the number of overlapping enriched terms between the algorithms being compared.

**Table 3.** Number of overlapping molecular function GO terms among the 5 algorithms.

	WGCNA	KINC	NetExtractor	EdgeCrafting	DGE_neg	DGE_pos
WGCNA	3					
KINC	14% (2)	13				
NetExtractor	8% (2)	2.7% (1)	24			
EdgeCrafting	6.7% (1)	18% (4)	23% (7)	13		
DGE_neg	0	0	20.8% (5)	12.5% (2)	5	
DGE_pos	9% (1)	22.2% (4)	3.1% (1)	4.7% (1)	0	9

**Table 4.** Number of overlapping biological process GO terms among the 5 algorithms.

	WGCNA	KINC	NetExtractor	EdgeCrafting	DGE_neg	DGE_pos
WGCNA	222					
KINC	41.5% (160)	323				
NetExtractor	47% (157)	28.3% (131)	270			
EdgeCrafting	40% (146)	46.1% (193)	48% (180)	288		
DGE_neg	7.8% (22)	0	16.3% (49)	6% (21)	79	
DGE_pos	25.3% (137)	39.3% (220)	19% (116)	28.7% (166)	0.1% (1)	456

**Table 5.** Number of overlapping cellular component GO terms among the 5 algorithms.

	WGCNA	KINC	NetExtractor	EdgeCrafting	DGE_neg	DGE_pos
WGCNA	40					
KINC	19% (10)	22				
NetExtractor	58% (29)	24% (12)	39			
EdgeCrafting	22% (12)	38% (13)	45% (20)	25		
DGE_neg	0	0	9.3% (4)	0	8	
DGE_pos	13.6% (12)	17.1% (12)	19.2% (16)	23.1% (16)	0	60

**Table 6.** Number of overlapping Pathway terms among the 5 algorithms.

	WGCNA	KINC	NetExtractor	EdgeCrafting	DGE_neg	DGE_pos
WGCNA	21					
KINC	29.5% (13)	36				
NetExtractor	20% (7)	1.7% (1)	21			
EdgeCrafting	3.5% (1)	7% (3)	7% (2)	8		
DGE_neg	0	0	29.6% (8)	0	14	
DGE_pos	5.4% (5)	13.2% (13)	0	3.8% (3)	0	75

**Table 7.** Number of overlapping disease terms among the 5 algorithms.

	WGCNA	KINC	NetExtractor	EdgeCrafting	DGE_neg	DGE_pos
WGCNA	49					
KINC	52% (39)	65				
NetExtractor	27.4% (17)	15% (13)	30			
EdgeCrafting	40% (37)	36% (38)	34.5% (28)	79		
DGE_neg	0	0	0	0	0	
DGE_pos	23.4% (38)	30.1% (50)	13.1% (21)	33% (57)	0	151

First, it can be observed that although we do not get a majority (over 50%) overlap in the actual list of genes as indicated by Table 2, we noticed that each gene list demonstrates functional enrichment even when accounting for a stringent significance threshold. This is an interesting observation since it signifies that different approaches to identify gene relationship result are unique set of genes that still independently demonstrate functional enrichment. Another interesting observation is that similar to the overlap of genes, NetExtractor and EdgeCrafting contain a significant amount of shared enriched terms, which is to be expected with the majority of the genes being common between the 2. It can also be observed that KINC and WGCNA share a high number of enriched terms. Although for the case of disease terms, EdgeCrafting demonstrated a high overlap of enriched terms with all other algorithms.

## Discussion

Functional analysis of genes detected from GCN construction algorithms crudely indicates the biological relevance signified by the network. Although in most cases it is essential to understand mechanism of action of the proposed network relationships, we restricted our analysis to preliminary biological indications reflected through functional MI enrichment. Considering that our aim was to compare the outputs of multiple algorithms, further biological investigation exceeds the scope of this report. To this end, we reviewed not only the genes and edges isolated by each algorithm (Table 1) but also the overlap of genes between each of them as represented in Table 2. This exercise in gene set comparison is not meant to determine the *superior* algorithm or objectively claim if 1 algorithm is objectively *better* than others based on a known ground truth, but rather to indicate that each algorithm is essentially designed to detect a specific type of relationship and may include edges that may be ignored by other algorithms. More specifically, this paper aims to propose that restructuring of input data into an alternative image-based format can detect biologically significant outputs that are comparable to previously implemented methods with the caveat that since each of those algorithms inherently are attempting to detect a different expression patterns, the networks constructed may have a limited overlap.

For example, WGCNA searches for linear pairwise correlations between all the samples within a particular gene–gene edge and, hence, shares a maximum overlap with KINC, which also restricts to the linearity of relationship. This would also explain why a relatively higher portion of genes are shared with NetExtractor and EdgeCrafting in cases where a linear relationship exists across all samples. A lower overlap for DGE-selected genes can be observed since the fundamental theory of bigenic, linear relationships does not hold true when monogenic condition-specific analysis is performed using DESeq2.

KINC, on the other hand, finds the optimal overlap with WGCNA as elaborated above, but the overlap of genes steeply declines when compared to other algorithms. We expected KINC to show a higher intersection with other algorithms including WGCNA, considering the fact that all of them account for condition-specific differential expressions. It was found, however, that KINC resulted in the most restrictive gene list due to the implementation of RMT to result in a scale-free network. KINC therefore shows poor correlation with the other algorithms, which output a more extensive gene lists.

EdgeCrafting, also modeled based on the theory of differential expression of subpopulations demonstrated by NetExtractor, exhibits a significant overlap of genes between the 2 algorithms (2,208 of 2,275 EdgeCrafting genes were in common with the NetExtractor output). This indicates that blob detection in image-based gene expression data can closely resemble the pipeline of GMMs followed by MI. Potential future work in this domain might include exploration of blob detection parameters to obtain a network most overlapping with that of NetExtractor.

We expect algorithms such as NetExtractor and EdgeCrafting to identify a larger subset of edges that depict multimodal expression patterns since the primary concept of their methodologies is to extend the scope of relationship that may exist and can be identified with the introduction of nonlinear metrics. An advantage of KINC is a granulated, significantly curated cluster of gene and their edges to investigate pathways of interest. On the other hand, NetExtractor and EdgeCrafting attempt to capture a larger number of gene pairs, which depict both linear and not necessarily linear relationships. This permits researchers to explore groups of genes and pathways that may not have been considered together previously.

The nonoverlapping enriched terms can also be interesting, suggesting that there are collective biological functions (Table 4), which are discovered by certain algorithms yet are completely missed by others. In particular, when observing Table 7 for disease terms, it was noticed that the highest overlap is still 52% between the 2 most similar algorithms with the common enriched terms reduced down when compared to others. Another observation is that the more commonly utilized DGE output in the positive and negative direction generated by DESeq2 resulted in a relatively large set of genes, which could be why functional enrichment, specifically for DGE_neg, results in very few enriched terms in most cases. These results indicate that most of these algorithms search for unique relationships within the same data while EdgeCrafting focuses on computationally scalable knowledge-independent detection of differential expression.

## Conclusion

In this report, we introduced the algorithm EdgeCrafting, where we integrate the theory of EdgeScaping and NetExtractor to isolate embedded, latent, nonlinear patterns for differentially expressed condition-specific tissue samples. This algorithm combines the image-based data conversion and nonlinear pattern detection of EdgeScaping as well as the ability to detect subpopulations within edges using GMMs followed by the nonlinear metric MI in NetExtractor to address the shortcomings of conventional GCN construction algorithms.

In EdgeCrafting, we convert gene expression data into an image format, which is dependent on 2 components: (1) the resolution of the bin size that does not change the inherent distribution of the data but only the resolution at which the patterns are observed, which was selected at 0.5, and (2) the orientation of gene *A*/gene *B*. As long as the axes of the genes remain consistent, i.e. gene A on *x*-axis while gene B on *y*-axis of the scatterplot, the image will always be consistent and reproducible. If the axes of genes A/B are flipped, a mirror image of the grayscale image will be obtained. This was a particular drawback in EdgeScaping since the orientation of the genes would determine the cluster the edge was assigned to. In EdgeCrafting however, this problem is addressed since the end goal is not to classify all edges into clusters but to determine differentially expressed nonlinear patterns within the samples of each edge. These patterns can be detected irrespective of the orientation of the genes on each axes.

We implemented EdgeCrafting at 3 different resolution of bin sizes: 1, 0.5, and 0.25, and selected 0.5 resolution for the best fidelity of data compression. Based on results of the isolated genes within each algorithm, it can also be observed that EdgeCrafting-detected genes that were significantly overlapping with NetExtractor, while KINC and WGCNA also shared a significant number of common genes.

## Data availability

EdgeCrafting source code and usage documentation are available at https://github.com/bhusain/EdgeCrafting under the MIT license and data files are available in figshare at https://doi.org/10.6084/m9.figshare.17701247.
